# To Link or Not to Link: Clause Chaining in Japanese Narratives

**DOI:** 10.3389/fpsyg.2019.03008

**Published:** 2020-02-07

**Authors:** Patricia M. Clancy

**Affiliations:** Department of Linguistics, University of California, Santa Barbara, Santa Barbara, CA, United States

**Keywords:** clause chain, Japanese, language development, semantic relations, switch-reference, narrative, mixed-effects model

## Abstract

Japanese is a clause chaining language, in which a sentence is formed by linking two or more clauses in a series (chain), using different forms of the verb in “non-final” clauses within the chain from the “final” clauses that end the chain. Since the verb comes at the end of a clause in Japanese, speakers must decide by the time they reach the verb at end of one clause whether or not to connect it to the following clause. This study analyzes the narratives of Japanese children and adults with the dual goal of discovering why narrators decide either to link the clause they are producing to the following clause or not to link, i.e., to end the clause chain. Stories were elicited from 60 3- to 7-year-old children and 10 adults, who performed two tasks: (1) telling the story depicted in a hand-drawn cartoon, and (2) viewing a 7-min video and then recounting the plot from memory. The data were analyzed qualitatively and quantitatively. The qualitative analysis examines story length and prompting, length of clause chains, variety of linking connectives, semantic relations—such as simultaneity, temporal sequence, and causality—between clauses in chains, shifts of subject from one story character to another (switch-reference), and contexts in stories where narrators end clause chains. For the quantitative analysis, a multifactorial, mixed-effects model was fit to determine which of several potential predictors have a significant effect on the probability that narrators will link clauses. The model is significant, though weak in discriminatory power. Among the significant effects, simultaneous relations between events increase the probability that narrators will link clauses, while changing the subject referent and reaching the end of a narrative unit, e.g., an episode, increase the probability that narrators will end their clause chain. There are no significant age effects: the children respond in the same way to the predictors as the adults. The qualitative and quantitative results of the study are interpreted with respect to the close relation between grammar (clause chains) and discourse (narrative structure) as well as the cognitive process of producing clause chains during narration.

## Introduction

Clause chaining occupies a key position at the nexus of clause-level grammar and discourse structure. From a grammatical perspective, a clause chain consists of one or more “medial” clauses with non-finite verbs preceding a “final” clause with a finite verb, while from a discourse perspective, clause chains may constitute or help construct discourse units of varying size and function (Longacre, 1985, pp. 264, 282–283). Since clause chains figure prominently in longer stretches of discourse, analyses of clause chaining have often focused on narratives.

Prior research on Japanese narratives has emphasized the function of clause chains in creating and managing discourse continuity (Iwasaki, 1993a; Watanabe, 1994). To create continuity, speakers link clauses into chains on the basis of semantic relations such as additive, sequential, causal, means, contrastive, concessive, and conditional (Hasegawa, 1996, pp. 176–210; Iwasaki, 2002, pp. 247–260). At points of discontinuity, speakers may choose grammatical forms that reflect and signal the break, e.g., ending a clause chain when switching the subject referent (Iwasaki, 1993a, pp. 75–76).

Switch-reference, i.e., referential discontinuity, has been a major focus of research on clause chains since many clause chaining languages have morphology encoding whether the subject referent will be maintained or changed in the following clause. This morphology serves the discourse function of assisting the listener’s referent tracking (Haiman and Munro, 1983, p. ix), which may be especially useful in narratives with multiple characters. Although switch-reference is not grammaticalized in Japanese, particular clause-linking connectives are followed by differing frequencies of switch-reference. Iwasaki (1993a, pp. 57–65) and Watanabe (1994, pp. 149–152) report that the most common connective, -*te* ‘and (then),’ is associated with subject continuity and -*tara* ‘when, if’ with discontinuity.

From a cognitive perspective, the process of producing of clause chains is organized by the typology of a language. Since Japanese is an SOV language and clause-linking forms either attach to the verb stem or follow an inflected verb, the speaker must decide by the end of each clause whether and how to link it to the following clause. This raises the question: What factors motivate speakers to link the current clause to the next or not to link, i.e., to end the clause chain in progress? The present study will address this question, using narrative data from Japanese children and adults.

### Clause Chaining and Monoclausal Verb Linking in Japanese

Japanese fits the definition of a clause-chaining language in that there is a clear distinction between the non-finite verb forms in medial clauses and the finite forms in the last clause of a chain. The default clause-linking -*te* suffix in (1a) contrasts with the past tense verb inflection -*ta* in (1b), which could be used to end a chain and determine the tense of the preceding medial clauses.


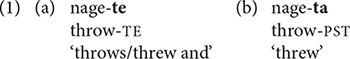


The verb suffix -*te* ‘and (then)’ is the most frequent clause-linking connective suffix and the most neutral semantically; in clause chains, the sequence of clauses connected by -*te* is generally taken to represent the sequence of events (Iwasaki, 2002, p. 261). Semantically more specific suffixes also appear on verbs in medial clauses, e.g., -*tara* ‘when, if’ and -*nagara* ‘while.’

Japanese clause chains depart from the standard definition, however, in that non-final clauses sometimes have finite verbs followed by a connective. Finite clauses with the conjunction *to* ‘when, if’ have received the most attention (Myhill and Hibiya, 1988; Iwasaki, 1993a, 2002). Since the verb preceding *to* is restricted to non-past tense, as in (2), it has been characterized as “partly non-finite” (Watanabe, 1994, p. 128); as with clause-linking suffixes, the actual tense depends on the final verb in the chain.


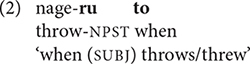


While connectives such as *to* ‘if, when’ have tense restrictions, many connectives occur with finite verbs inflected for either past or non-past tense, as in (3).


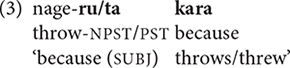


In discourse, Japanese speakers freely link clauses with non-finite (1a), “partly finite” (2), and fully finite (3) verbs within the same clause chain (Alpotov and Podlesskaya, 1995, p. 482; Iwasaki, 2002, p. 265). For purposes of this paper, therefore, I will use the term “non-final” rather than “medial” for clauses other than the last one within a chain. Despite their grammatical differences, non-final clauses typically display similar particles with pragmatic functions and prosodic patterns.

In addition to clause-final connectives, Japanese also has several lexical adverbs with a linking function. These connectives, such as *soshite* ‘and then’ and *sorede* ‘and so/then’, are usually found at the start of a new clause. Thus two linked clauses often feature a connective verb suffix such as -*te* at the end of the first clause as well as a freestanding adverbial like *sorede* at the beginning of the second clause. In this paper, I will focus exclusively on the former, clause-final type of clause linking.

The -*te* form in (1a) also serves a variety of monoclausal functions in which -*te* is suffixed to the stem of the initial, main verb while the second verb functions as an auxiliary. For example, V-*te* plus *(i)ru*/*aru* ‘exist’ forms progressive, perfective, and resultative aspects; V-*te* plus *kuru* ‘come’ and *iku* ‘go’ encodes inchoative aspect from different points of view; V-*te* plus *shimau* ‘put away’ (contracted to V-*chau* in Tokyo dialect) conveys completive aspect; and V-*te* plus *ageru*/*kureru*/*kudasaru* ‘give’ and V-*te* + *morau* ‘receive’) form benefactive constructions (Hasegawa, 1996; Iwasaki, 2002).

### Acquisition of Monoclausal V-*te* V and Clause Chaining

Research on the acquisition of Japanese has documented the emergence of -*te* in monoclausal and clause-linking functions at approximately 2;0–2;6 years of age [summarized in Clancy (1985, pp. 425-426, 471-475)]. V-*te*, historically a reduced form of the benefactive construction V-*te kure*/*kudasai* ‘V-TE give.IMP,’ is a very common imperative in child-directed speech. In the first few months of inflecting verbs, children use -*te* in this function productively and contrastively with inflections such as -*ta* (past) and -*u* (non-past) and aspectual forms incorporating -*te*, such as V-*teru*/-*teta* (non-past/past progressive or resultative).

V-*te* also appears early in constructions expressing deontic modality, including requests, suggestions, permission, and prohibition. The main verb, inflected with variants of the -*te* suffix or -*tara* ‘if,’ is followed by predicates such as *ii* ‘is all right’ or *dame* ‘not good’, which take the main clause as their argument (Hasegawa, 1996, pp.151–156). In the game of pretend play in (4), the child is telling her mother to eat the imaginary food she is serving (Akatsuka and Clancy, 1992, p. 186). The child’s age appears in parentheses.


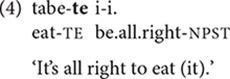


Linking clauses with -*te* also begins soon after 2 years of age. In (5), the same child as in (4) is explaining how to place one game piece on top of another. She uses -*te* to link the non-final clause to the final clause, which has an inflected verb plus the sentence-final particle (SFP) *no* (Clancy, 1985, p. 472).


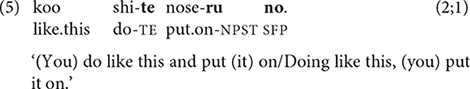


Following -*te*, several other connectives appear in two-clause chains during the first half of the third year. Okubo (1967) documents the following forms in her daughter’s speech before 2;6 years of age: *kara* ‘because,’ -*tara* ‘when, if,’ -*tari* ‘and’ (for repeated or representative actions), *toki* ‘when,’ *to* ‘when/if,’ *noni* ‘although’ and *kedo* ‘but.’ The suffix -*nagara* ‘while’ did not appear until much later, at 3;8.

Between 2 and 3 years of age, benefactive constructions with -*te* are also acquired (Horiguchi, 1979). Takahashi (1975) reports that 4-year-old children have acquired most constructions involving deictic and metaphorical motion with V-*te kuru/iku* ‘V-TE come/go’ (1975, cited in Hasegawa, 1996, p. 109).

In sum, longitudinal studies have found that by 3 years of age, Japanese children are using V-*te* as an imperative, in various tense/aspect forms incorporating -*te*, and in several constructions in which the main verb with -*te* suffix is followed by an auxiliary verb. In addition, the basic tools for clause chaining are in place: children can use -*te* and several other connectives to link clauses, and they know how to end clause chains with inflected verbs and, optionally, sentence-final forms such as *no*.

### The Present Study

The primary goal of this study is to examine the semantic and discourse properties of clause chains in Japanese narratives, focusing on the factors that motivate narrators to link clauses into chains and to end clause chains in progress. Qualitative analysis will be used to address the following questions: When do children begin producing clause chains in narratives, and how do their clause chains change over time? What semantic relations can be identified in the clause chains of young children and adults, and which connectives do they use in conveying those relations? Does switch-reference impact narrators’ choice of the default -*te* clause-linking suffix vs. the semantically more specific -*tara* ‘when,’ as documented in prior research? What discourse contexts are associated with narrators’ use of finite verbs to end clause chains? Once potential motivations for clause linking and chain ending have been identified through qualitative analysis, a statistical analysis will be employed to discover which ones have a significant impact on narrators’ decision to link—or not link—the clauses in their stories.

The paper is organized as follows. The following section describes the methodology for the study. The section “Qualitative Analysis” presents an analysis of the narrators’ clause chains, with examples illustrating their key properties. In the section “To Link or Not to Link: A Statistical Analysis,” several potential predictors of clause chaining vs. chain ending are modeled in a multifactorial, mixed-effects analysis. The final section concludes the paper with a general discussion and suggestions for further research.

## Methodology

The narratives for this study were elicited using two methods: (1) having narrators tell the story of a nine-frame, hand-drawn cartoon, and (2) having narrators view a short video and then recount the story from memory. The first method has been popular in developmental research, e.g., Berman and Slobin’s (1994) use of a wordless picture book about a runaway frog to elicit a multi-language corpus of “frog stories.” The video method was introduced by Chafe (1980a) in his Pear Story project; participants viewed a short film and then told the story to an interviewer. Both methods have the advantage of giving the analyst full access to the experience underlying the story. Since narrators are all telling the same story, common patterns are easily recognizable, while comparison of adult vs. child narratives helps identify developmental differences.

### Subjects

The participants in this study were 60 children and ten adults living in a middle-class suburb of Tokyo. The children, five girls and five boys at each of the age ranges in Table 1 were attending either a private kindergarten for 3- to 6-year-olds or the first grade of a public elementary school. The adults were undergraduate and graduate students of 21–29 years old who were attending a private university nearby.

**TABLE 1:** Cartoon and video data.

**Age**	**Cartoon stories**	**Clauses**	**Video stories**	**Clauses**
3;8 – 4;0	9	111	10	152
4;4 – 4;8	10	103	10	110
5;0 – 5;4	10	108	10	332
5;8 – 6;0	10	95	10	245
6;4 – 6;8	9	108	10	363
7;0 – 7;4	8	114	10	328
Adult	10	236	10	755
		875		2,285

### Materials

The cartoon used to elicit narration depicts in nine frames a story featuring two girls at a playground (see Appendix). During elicitation, the frames were displayed (unnumbered) in transparent covers taped horizontally from left to right. The video is a 7-min segment titled *“Akachango”* ‘Babytalk’ from the *Sazaesan* television series, a popular program about the everyday life of Sazaesan and her family. Since this program was being televised at the time of the study, the narrators were familiar with the characters and typical storylines. The cartoon and video stories are summarized below.

Cartoon frames: (1) An empty playground in a park. (2) Two girls, identified as Yukiko and Sachiko before the cartoon was shown, arrive at the playground, holding hands with their mothers and carrying balloons. (3) Sachiko is on a swing, holding her balloon. (4) Yukiko is on the slide, holding her balloon. (5) Yukiko is halfway down the slide, with a startled expression, as her balloon flies away. (6) Yukiko looks downcast as Sachiko approaches, holding her balloon. (7) Sachiko gives Yukiko her balloon. (8) Yukiko gives Sachiko her hat. (9) The girls leave the playground with their mothers, Yukiko holding Sachiko’s balloon and Sachiko wearing Yukiko’s hat.

Video: Baby Ikura is being cared for at Sazaesan’s house because his mother is ill. He tears the house apart, opening drawers and throwing the contents around, to the great annoyance of Sazaesan’s younger siblings, Wakame and Katsuo. The next day, Ikura’s father returns to say that the baby will be at home with his now-recovered mother; then he apologizes for Ikura’s bad behavior. Sazaesan’s family is mystified, since they had not complained and Ikura does not know how to talk. Later, however, Ikura’s mother reports that he told her about having searched Sazaesan’s house for medicine to give her. She explains that a mother can understand her baby, which she proves by successfully “translating” Ikura’s babbled claim that he has seen a snail on a leaf.

Table 1 summarizes the data for this study, including the number of cartoon and video stories available for analysis, as well as the total number of clauses they contain.

Of the 3,160 clauses in Table 1, 23.2% consist of single-clause sentences. The remaining 2,428 clauses occur in sentences having at least two clauses, and provide the data for the statistical analysis. The overwhelming majority of these clauses appear either in chains having only non-finite verbs in their non-final clauses (58.7%) or in chains with both non-finite and finite verbs in their non-final clauses (35%). Only 6.3% appear in chains having only finite verbs in their non-final clauses.

### Data Collection and Transcription

The elicitation sessions took place at the kindergarten and elementary school attended by the children. Each child met individually with two female college students, who served as elicitor and listener for the storytelling tasks. The cartoon elicitation was presented as a game in which the child sat beside the elicitor, with the cartoon spread out on a table in front of them, and told the story to the listener, who sat with her eyes covered to reduce the child’s reliance on pointing. Next, the listener made an excuse to leave the room, and the child watched the Sazaesan video with the elicitor. The listener then returned and asked the child: *Donna hanashi datta no?* ‘What was the story?’ If the child fell silent during narration, the elicitor prompted: *Sorede*? ‘And then?’ or *Doo shita no*? ‘What happened/what did s/he do?’ If the child still could not proceed, the elicitor used a standard set of specific prompts for different points in the storyline. If verbal prompts failed, the child was shown a few photos depicting scenes from the video. The same procedure was followed with the adults, except that no prompts were used and the listener did not cover her eyes during the cartoon elicitation.

The narratives were transcribed drawing on the conventions in Chafe (1980a, p. xv) and elaborated in Du Bois et al. (1992, 1993): commas mark the ends of intonation units and periods represent sentence-final falling intonation, regardless of whether sentence-final syntactic closure was used. Although most intonation units are preceded by silent pauses, they are not included here, in order to facilitate the reading of examples. Japanese narrators, especially children, often end non-final clauses and clause-internal intonation units with the tag-like particle *ne* and rising intonation (Clancy, 1982, pp. 61–63; Iwasaki, 1993b). In an extended turn such as a narrative, *ne* seeks the listener’s continued cooperation in holding the floor (Cook, 1992, p. 524). Final clauses in a chain typically end with a verb inflected for past tense, the SFP *no*, and falling intonation.

## Qualitative Analysis

In this section, a qualitative analysis will be presented of the following properties of the narratives: story length and prompting, semantic relations in clauses linked by the three most common connectives in the children’s stories, switch-reference in non-final clauses, reformulations that change a connective or the status of a clause as non-final/final, chain length and diversity of linking forms, and narrative contexts associated with the end of clause chains.

### Story Length and Prompting

Table 2 summarizes the narrators’ average story length and percentage of prompted clauses at each age. The length of the children’s cartoon stories is fairly constant, but beginning at 5 years of age there is a marked increase in the length of their video stories. The adults’ stories, especially their video stories, are much longer on average than the children’s. The greater length of the video stories suggests that the complex plot afforded a more open-ended opportunity for narration.

**TABLE 2:** Story length and frequency of prompting.

**Age**	**Mean # of clauses**		**Mean % of clauses**	
	**per story**		**prompted**	
	**Cartoon**	**Video**	**Cartoon**	**Video**
3;8 – 4;0	12	15	0.32	0.70
4;4 – 4;8	10	11	0.39	0.44
5;0 – 5;4	11	33	0.05	0.12
5;8 – 6;0	10	25	0.08	0.11
6;4 – 6;8	12	36	0.03	0.06
7;0 – 7;4	14	33	0.02	0.08
Adult	24	76	0	0

The children’s need for prompting decreases dramatically with age, as Table 2 shows. While 32% of the 3-year-olds’ cartoon clauses and 70% of their video clauses need prompting, by 5 years of age, the children are much more capable of narrating without assistance. Overall, the video stories require more prompting; even the adults occasionally commented on their difficulty recalling the complicated storyline.

The stories of children who need extensive prompting take the form of a question-answer dialogue, similar to the heavily scaffolded conversational stories of 2-year-olds (Miller and Sperry, 1988). These dialogic narratives often consist entirely of single-clause responses, with no clause chains. The story excerpted in (6) includes 17 short sentences with 15 interviewer prompts. (The participant number and age of narrator appear in parentheses; I = interviewer, C = child).


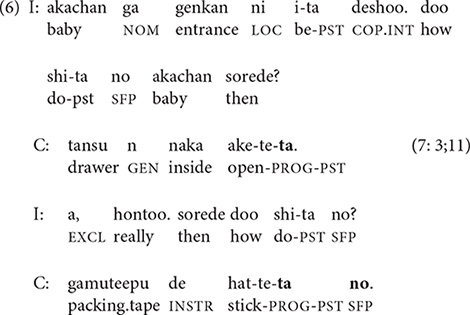


While most 3-year-olds need continuous scaffolding of their video narratives, two told the story with minimal or no prompting. The video story excerpted in (7) is 34 sentences long and has only a single prompt at the start, which is ignored. Nevertheless, as in (6), the narrator relies almost exclusively on single-clause sentences.


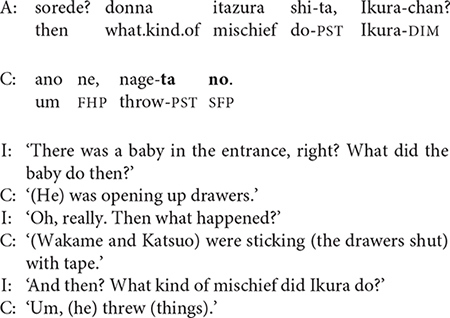


Like the narrators in (6) and (7), many 3-year-olds formulate their stories one clause at a time with almost no clause linking; this pattern essentially equates clause and sentence.

### To Link: Semantic Relations in Non-final Clauses

#### Clause Linking With -*te*

Although some children under 5 years of age make minimal use of clause chains in their stories, others freely use -*te* ‘and (then),’ and sometimes more specific connectives such as -*tara* ‘when,’ to create chains of two or more clauses. The non-final clauses in these chains feature many of the semantic relations described in research on Japanese adults’ clause chaining (Watanabe, 1994; Hasegawa, 1996).

Three-year-olds, for example, often use -*te* in cases of simultaneous or temporally overlapping relations between two linked clauses. Such clauses are typically characterized as conveying manner, i.e., the way in which the action is performed (Watanabe, 1994, p. 132; Genetti, 2005, pp. 53–54). The narrator in (8) is recounting the arrival at the playground of the two girls in the cartoon story.


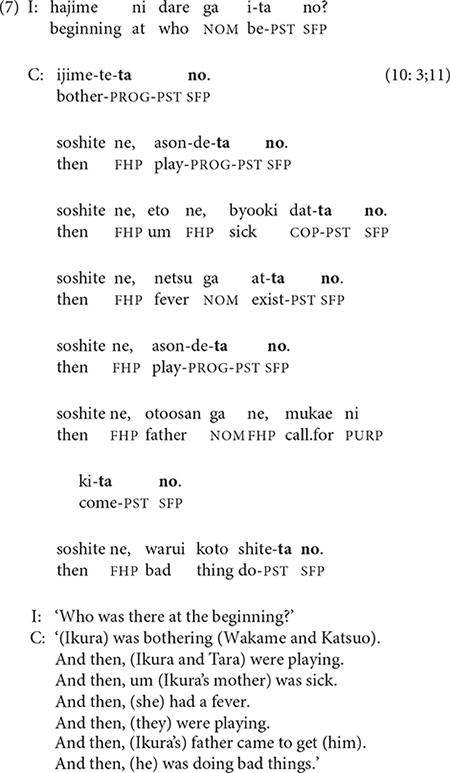


In this example, there is potential ambiguity between a monoclausal interpretation of V-*te kuru* ‘V-TE come,’ in which *mot-te kita* ‘carry-and came’ is lexicalized as ‘brought’ (‘they brought balloons’) and a biclausal, clause-linking interpretation (‘holding balloons, they came’). A number of criteria were used to differentiate between monoclausal and biclausal cases. When only a single event was involved, e.g., *booshi kashi-te ageta* ‘lend-and gave hat’ (lit. ‘gave the favor of lending (her) hat’) or *fuusen ton-de itta* ‘balloon fly and went’ (‘the balloon flew away’), a monoclausal interpretation was assigned. When a two-verb sequence could be interpreted as involving either one or two events, additional factors were taken into account. If the two verbs were articulated in separate intonation units, they were coded as comprising two clauses. A biclausal interpretation was also made if lexical material separated the two verbs; for example, in *fuusen motte mama to kita* ‘holding balloons with their mothers they came’, the phrase *mama to* ‘with (their) mothers’ intervenes between *mot-te* ‘carry-and’ and *kita* ‘came,’ ruling out a monoclausal reading of *motte kita* as ‘brought.’ In many cases, context distinguished between monoclausal vs. biclausal interpretations, making one of the interpretations implausible given the relevant events in the cartoon or video.

Using these criteria, only a handful of ambiguous cases remained, such as (8), in which both monoclausal and biclausal interpretations are plausible. Since the arrival of the main characters at the empty playground is the key event of the second frame of the cartoon and was explicitly mentioned by most narrators, (8) was coded as biclausal, although this interpretation is challengeable. When the narrator of a cartoon story had already mentioned the girls’ arrival at the park, or when the focus was exclusively on the balloons, the sequence *mot-te kita* ‘carry-and came’ was analyzed as monoclausal: ‘they brought balloons.’

The most common type of simultaneous or partially overlapping relation between clauses involves the use of the quotative particle *tte* to convey speech that is produced while performing an action, as in (9).


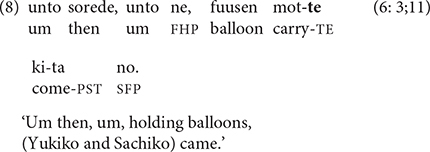


Since the two-verb sequence *yut-te moratta* ‘say-and received’ in (9) has the same form as a benefactive construction, the sequence in (9) could be interpreted as ‘(Sachiko) received the favor of (Yukiko) saying “thank you.”’ However, context clearly rules out this monoclausal interpretation of the eighth cartoon frame, in which Yukiko gives Sachiko her hat (see Appendix).

The quotative construction in (9) is frequently used without an overt verb of saying, which leaves the clause with the reported speech ending in the quotative particle *tte*, as in (10).


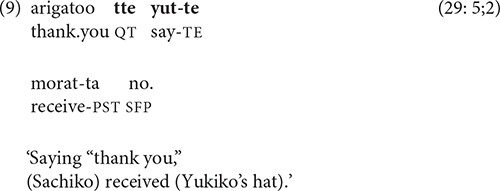


For purposes of this paper, a clause ending in *tte* is treated as a non-final clause when produced with non-final intonation and as a final clause when used with sentence-final intonation.

Temporal sequence is generally regarded as the unmarked relation between clauses in a chain, whose order cannot be reversed without changing the meaning (Iwasaki, 2002, p. 261). In (11), for example, the reverse order of clauses does not make sense.


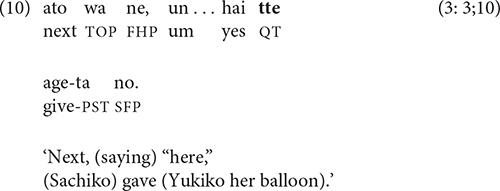


In addition to temporal relations of simultaneity and sequentiality, clause-linking with -*te* frequently conveys causality, as in (12).


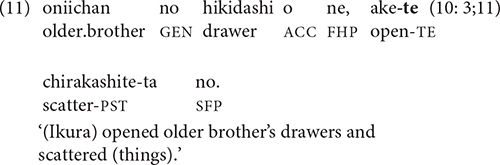


The sequence of events in the video that was most likely to elicit clause chaining in the youngest children’s stories is the one in (13):


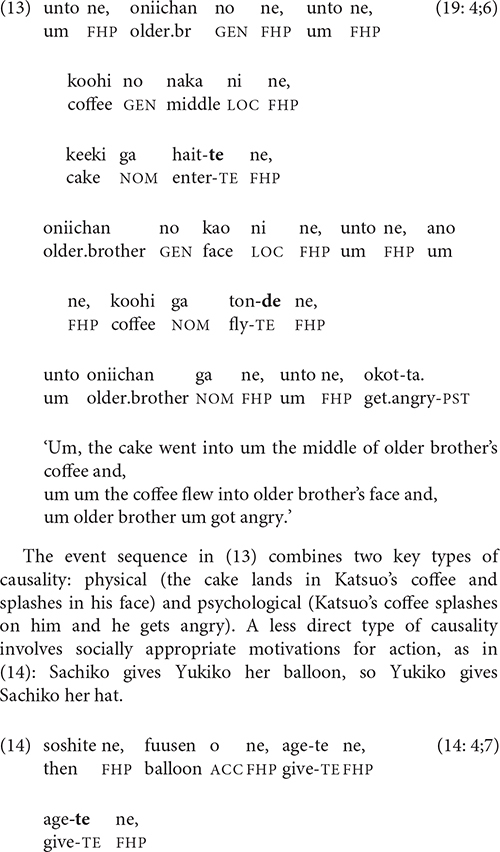


The event sequence in (13) combines two key types of causality: physical (the cake lands in Katsuo’s coffee and splashes in his face) and psychological (Katsuo’s coffee splashes on him and he gets angry). A less direct type of causality involves socially appropriate motivations for action, as in (14): Sachiko gives Yukiko her balloon, so Yukiko gives Sachiko her hat.


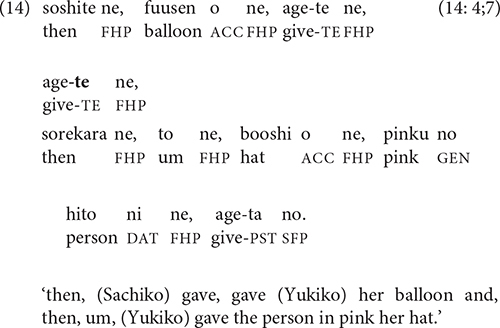


Clauses that compare two states, events, or situations are also sometimes linked by -*te*. The semantic relation in (15) is contrastive, while that in (16), which first appears in the 5-year-olds’ stories, has been termed “additive” [Hasegawa, 1996, p. 6; cf. Longacre’s “coupling” relation (Longacre, 1985, p. 241)].


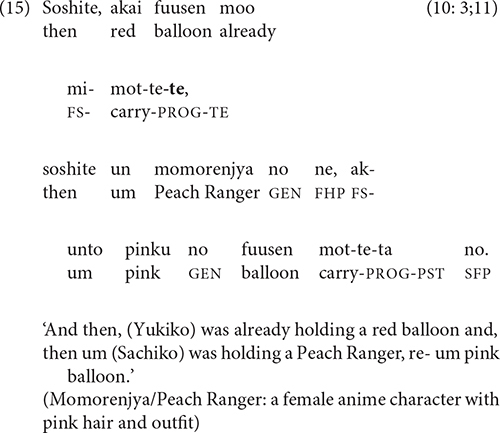



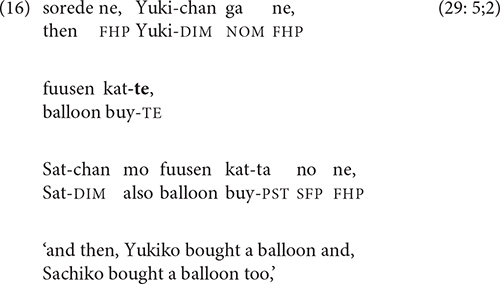


#### Clause Linking With *Kara* ‘Because’ and -*Tara* ‘When’

As examples (8–16) illustrate, -*te* is compatible with a wide range of semantic relations. V-*te* is the most common connective, appearing in 77% of the children’s non-final clauses and 46% of the adults’. Next most frequent for the children are -*tara* ‘when’ (7%) and *kara* ‘because’ (5%). *Kara* ‘because’ first appears in the stories of the 3-year-olds, as in (17).


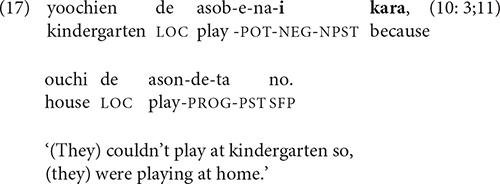


Although -*te* can be used to link a reason to an action or event, it is much more common for narrators of all ages to use *kara* ‘because’, as in (18).


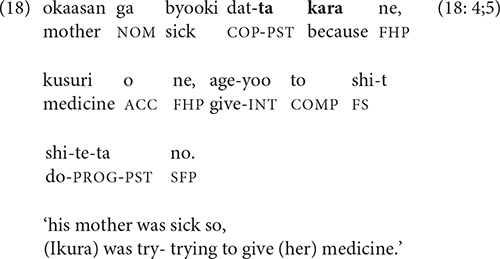


The suffix -*tara* ‘when’ first appears in the stories of the 4-year-olds. Since -*tara* can serve many of the same temporal functions as -*te* but is much less frequent, it breaks the monotony of -*te* linking, creating a tighter sense of interclausal connection.

Hasegawa (1996, pp. 208–209) includes “setting” as one of the functions of -*te* linking; in these stories, settings often present the arrival of new characters with -*te*. But using -*tara*, as in (19), evokes a sense of closer relation to, and expectation about, the events that are to come.


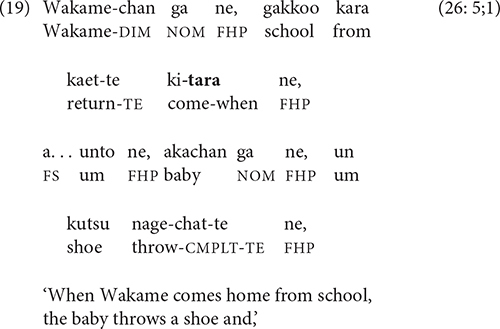


An especially common use for -*tara* is to create a tight link between successive turns in reported dialogue, such as the question-answer sequence in (20).


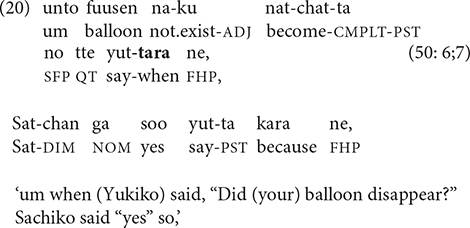


The sense of something tellable to come that -*tara* conveys can be used by narrators to increase the suspense at key moments in a story. In the video scene that elicited -*tara* most reliably, Ikura’s mother declares that her babbling child has said that there is a snail on a leaf, and Wakame and Katsuo rush to discover whether this is true.


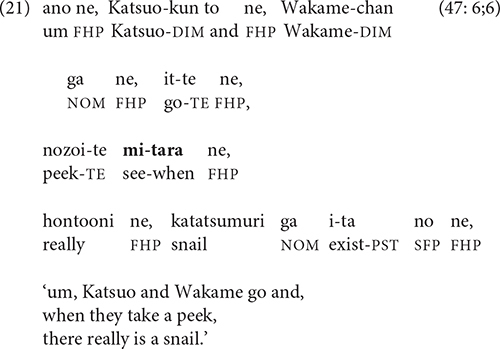


Iwasaki describes this use of -*tara* as comprising a voluntary action followed by the stative perception it enables (Iwasaki, 1993a, p. 71), which Watanabe characterizes as the “discovery construction” (Watanabe, 1994, p. 172).

-*Tara* can also have a concessive flavor, which Watanabe dubs the “action in vain” interpretation (Watanabe, 1994, p. 136). In (22), Wakame and Katsuo tape their desk drawers shut in anticipation of Ikura’s return, only to discover that he has not come.


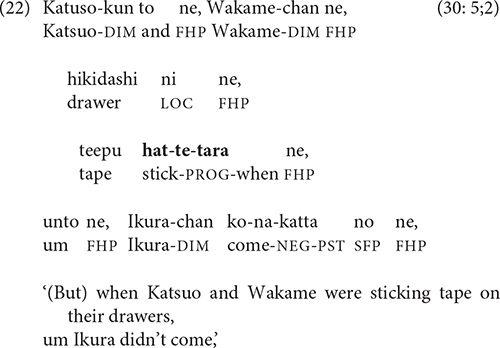


The conjunction *to* ‘when, if’ is extremely rare in the children’s stories, although the adults occasionally use *to* instead of -*tara* in the contexts in (19–22). Watanabe’s “discovery” and “action in vain” analyses are based on *to* but are equally applicable to -*tara* in the present data. Presumably, the children will begin to use *to* for certain -*tara* functions at a later stage of development.

Examples (8–22) illustrate the most common semantic relations between non-final clauses in the children’s stories. The statistical analysis of predictors that increase/decrease the probability of clause linking will include the following semantic relations: manner, temporal sequence (with ‘dialogue’ as a subtype), causality, setting, and comparative (defined as encompassing additive, contrastive, and concessive relations).

### Switch-Reference in Non-final Clauses

As noted in the Introduction, research on Japanese clause chains has found differing frequencies of switch-reference following different connectives. For example, Iwasaki reports that in his sample of spoken narratives, 89% of -*tara* clauses but only 19% of -*te* clauses were followed by switch-reference (Iwasaki, 1993a, p. 64). Myhill and Hibiya found a similar pattern (66% switch-reference following *to* vs. 27% following -*te*) in a novel (Myhill and Hibiya, 1988, pp. 377, 390), and Watanabe in narratives from elementary school textbooks: 73% switch-reference after *to*, 17% after -*te* (Watanabe, 1994, p. 150). Iwasaki concludes that -*te* is used for continuous subjects and -*tara* for discontinuous subjects, while Watanabe proposes that -*te* is a “true Same Subject device” in clauses with human subjects, 100% of which maintained the same subject referent in the clause following -*te* (Watanabe, 1994, p. 152).

The cartoon and video in this study feature multiple characters engaged in action-reaction scenarios and back-and-forth dialogue. The cartoon, in fact, was specifically designed to elicit switch-reference, and many cartoon stories, such as (23), have a different subject in each clause. In (23), the narrator switches the subject referent following both -*te* (lines 2, 5) and -*tara* (line 3).


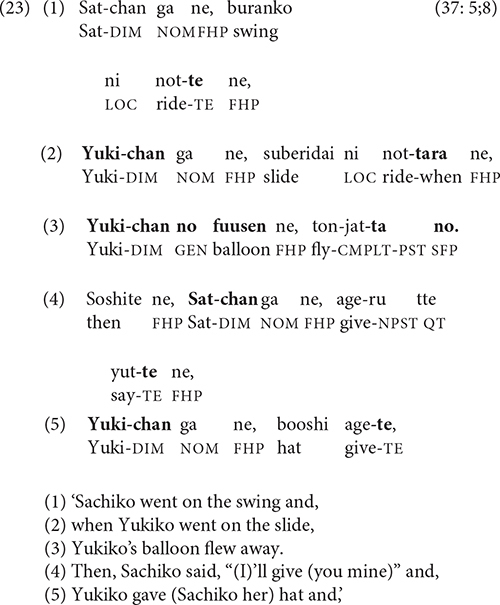


Table 3 compares the percentage of switch-reference (DS = different subject) following -*te* ‘and (then)’ and -*tara* ‘when’ in the cartoon vs. video narratives. In the cartoon stories, as well as the video stories of children under 5 years of age, -*tara* is too infrequent to permit reliable conclusions. However, the expected distinction between -*tara* and -*te* is evident in the video stories of the children over 5 years old, who averaged 80% switch-reference following -*tara* vs. 53% following -*te*. The adults’ video stories show a similar pattern: 81% switch-reference after *tara*, 46% after -*te*.

**TABLE 3:** Switch-Reference following -*te* ‘and (then)’ vs. -*tara* ‘when’.

**Age**	**Cartoon Stories**				**Video Stories**			
	**-*te***		**-*tara***		**-*te***		**-*tara***	
	***N***	**% DS**	***N***	**% DS**	***N***	**% DS**	***N***	**% DS**
3;8 – 4;0	28	0.64	0		19	0.58	0	
4;4 – 4;8	22	0.73	4	1.00	33	0.52	4	0.75
5;0 – 5;4	54	0.74	2	1.00	135	0.49	26	0.81
5;8 – 6;0	44	0.82	1	1.00	121	0.50	10	0.70
6;4 – 6;8	55	0.75	1	1.00	212	0.58	19	0.84
7;0 – 7;4	71	0.65	5	0.80	184	0.55	18	0.83
Adult	83	0.33	3	0.33	200	0.46	32	0.81

While these findings support the claim that -*tara* is more strongly associated with switch-reference than -*te*, the percentage of switch-reference following -*te* is much higher than in prior studies: combining all age groups, the children average 72% switch-reference after -*te* in their cartoon stories and 54% in their video stories. Even the adults’ lower frequencies (33% and 46%, respectively) exceed the 17–27% rates of switch-reference following -*te* in previous research.

Apparently, the rate of switch-reference varies considerably depending on the storyline, and these stories call for frequent changes of subject. The narrator’s degree of elaboration and point of view are also relevant. Compared to the children, the adults tend to maintain a single character’s point of view in their cartoon stories and to recount the actions of each character in greater detail before switching subject, resulting in a lower percentage of switch-reference.

Given the frequency of switch-reference with -*te* in these narratives, which feature human subjects in most clauses, it is clear that -*te* is not functioning as a Same Subject device. Nevertheless, consistent with previous findings, -*tara* is more likely than -*te* to be followed by switch-reference, setting up expectations that probably guide narrators’ choice of connective and offer listeners some assistance in referent tracking.

### Reruns: Linking and Delinking Reformulations

Narrators’ process of deciding whether to link—or not link—adjacent clauses is usually invisible. Such decisions are subject to monitoring, however, and occasionally are revised. In (24), the narrator introduces the setting for a new episode with -*tara* and then switches to -*te*.


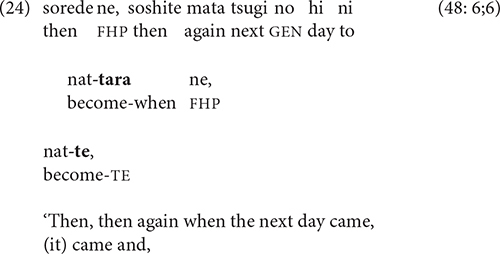


Such reruns are not always self-corrections; narrators sometimes use a rhetorical strategy of “recapitulation” (Genetti, 2005, pp. 49–50), in which the final clause of one chain is reformulated as the initial clause of the next [see Guérin and Aiton (2019) on “bridging constructions.”] In (25), Wakame and Katsuo’s question in line 1 is the final event of one episode; initially presented as the final clause in a chain, it is then reiterated in line 2 in shortened form as a connecting link to the first event of the next episode, which starts in line 3.


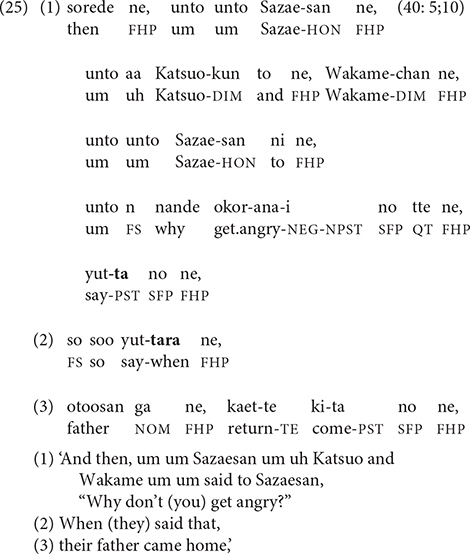


The recapitulation strategy reformulates a final clause as a non-final one. Alternatively, as in (26), the narrator may delink a non-final clause, reformulating it as a final clause.


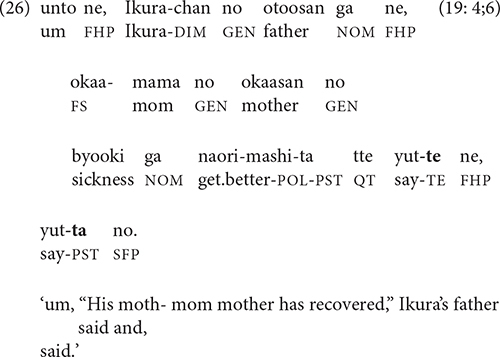


Unlike the clauses in the preceding section, reformulations do not have a particular semantic relation to the preceding clause; instead, they simply repeat, elaborate, or summarize the clause they follow [cf. Longacre’s “paraphrase” relation (Longacre, 1985, pp. 246–247)]. This type of interclausal connection, here termed “rerun,” will be included in the statistical analysis along with the semantic relations from Section “To Link: Semantic Relations in Non-final Clauses.”

### Chain Length and Diversity of Connectives in Non-final Clauses

Despite the minimal use of clause chains by many children under 5 years of age, five narrators use four- to six-clause chains, and one 3-year-old uses a single, 20-clause chain to tell the entire cartoon story. The narrative in (27) illustrates this ‘story-in-one-chain’ pattern. (The accent marks represent heavy stress and high pitch, a prosodic scheme sometimes used at the ends of intonation units instead of *ne*).


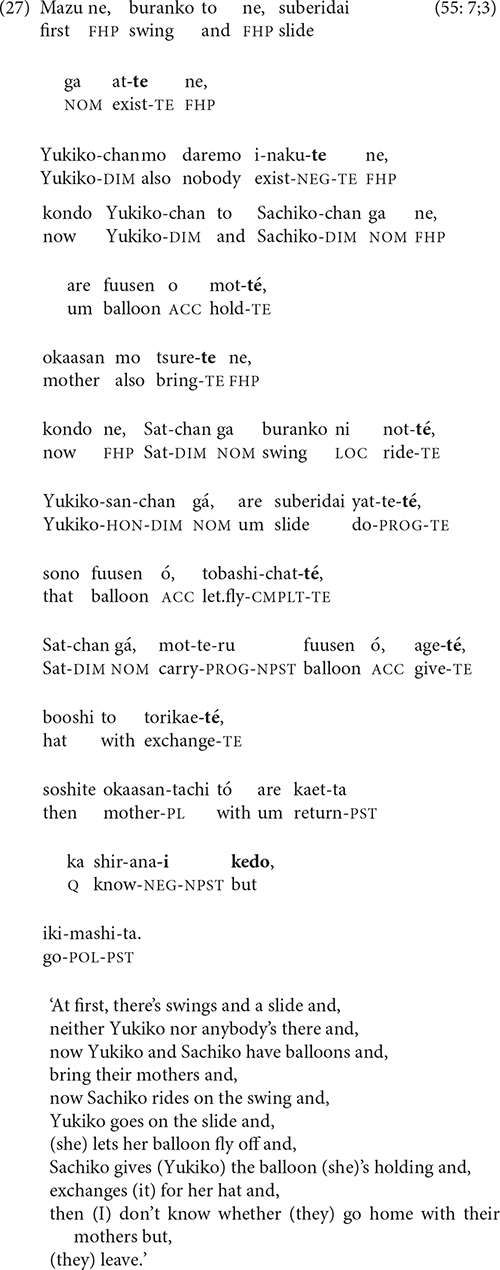


Narrating an entire story in a single clause chain seems at first glance to be the polar opposite of the one-clause-at-a-time pattern in stories with no clause chains. In a sense, though, the two patterns are similar: both create a narrative with no multi-clause prosodic and grammatical unit that is more inclusive than a single- clause sentence but less inclusive than a sentence composed of the entire story. This kind of intermediate-sized, multi-clause unit is exactly what clause chains can help create within a narrative. None of the adults recount an entire narrative, not even the cartoon story, in a single clause chain.

The length of clause chains varies considerably, not only across different speakers but within a single story. Narratives with one or two extremely long chains also usually include chains of more moderate length. For example, one 6-year-old whose video story has a 25-clause chain also includes six two- to five-clause chains.

The diversity of connectives in non-final clauses also varies a great deal. Very long chains typically rely on -*te*, as in (27), but sometimes even young children’s chains are both extremely long and internally diverse. The video story of one 4-year-old, for example, includes a 35-clause chain with 26 -*te* connectives, as well as -*tara* ‘when,’ *kara* ‘because,’ -*tari* ‘and’ (for representative actions), and *mae(ni)* ‘before.’

Chain length and the diversity of connectives in non-final clauses (Diverse Non-final) will be included in the statistical analysis to determine whether they exhibit developmental trends and/or impact the probability of clause linking.

### …or Not to Link: Ending Clause Chains

Turning from non-final to final clauses, this section will consider two discourse contexts, switch-reference and the boundaries of narrative units, where narrators sometimes end their clause chains.

#### Switch-Reference in Final Clauses

Research on Japanese clause chains has identified switch-reference as a site where narrators sometimes end clause chains. In his analysis of third-person subjects in adults’ narratives, Iwasaki found that 77.3% of clauses end with a finite verb when the next clause has a different subject referent, as compared with only 32.5% when the subject referent is the same (Iwasaki, 1993a, p. 76).

This pattern can be seen in (28): at the end of line 3, the narrator ends the clause chain in progress with a finite verb and SFP *no* before changing the subject referent from Ikura to Katsuo and Wakame in line 4.


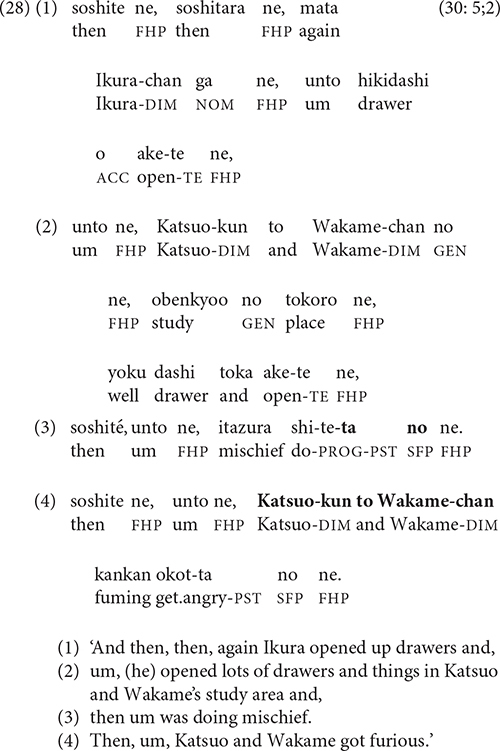


The role of clause chains in creating episode-internal sub-units is apparent in (28). By ending the first clause chain at the point of switch-reference, the narrator organizes the events of the episode into an Action (lines 1–3) – Reaction (line 4) pattern.

#### Boundaries of Narrative Units

Switch-reference creates a break in the referential continuity of a story, but usually a relatively minor one: the narrator’s focus simply shifts from one story character to another, often within the same episode, as in (28). The boundaries of major discourse units, on the other hand, represent a more significant break in narrative continuity. Chafe has characterized episode boundaries as places of scalar change co-occurring along one or more dimensions including space, time, character configuration, and orientation to a new central event (Chafe, 1979, pp. 176–180; Chafe, 1980b, pp. 40–47). The narrator typically presents such changes in the setting of a new episode; the number of changes and their degree of elaboration help create episode boundaries of varying degrees of strength. The cognitive impact of these combined changes in time, location, characters, and action is evident in the location of recall problems. In the video stories, if a narrator experiences memory failure, it is usually at an episode boundary. It makes sense, then, that a key site for ending a clause chain is at the boundary of an important narrative unit.

In (29), the narrator begins by presenting the setting for her cartoon story—the playground, arrival of characters, and key props (balloons)—in lines 1–3. She ends her first clause chain at the end of the setting in line 3, with a finite verb and the SFP *no*. Then lines 4–6 present the events of the first episode, in which the girls play and Yukiko’s balloon flies away.


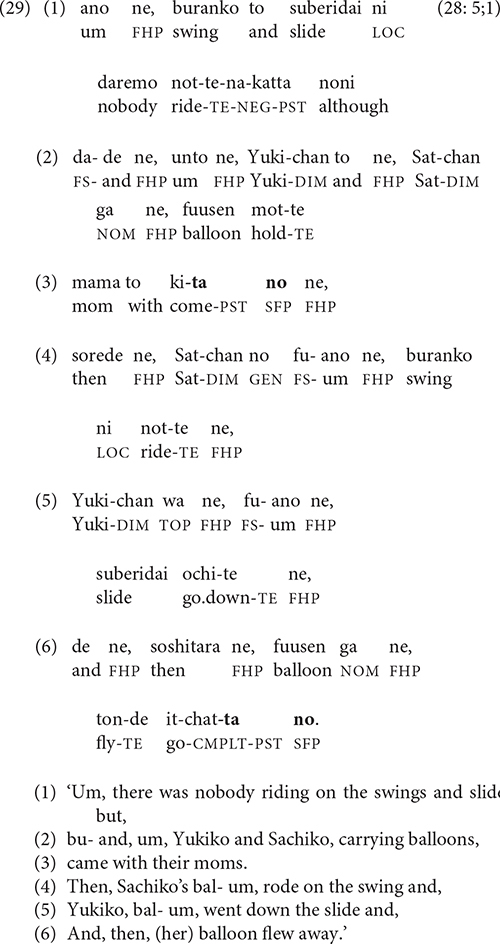


After ending her second clause chain in line 6, the narrator proceeds to the next episode. Thus the ends of clause chains in (29) not only reflect changes in story content but help create boundaries between narrative units.

Another type of boundary involves a shift in the narrator’s point of view. The Sazaesan video, in which the story characters have differing amounts of information from one another—and from the narrator—often elicited this type of shift, as in (30). In lines 1–3, the narrator is recounting the episode during which Wakame and Katsuo discover the snail on a leaf [see example (21)]; at the end of line 3, she concludes a 23-clause chain with this discovery. Then, instead of explaining from the characters’ perspective how Sazaesan’s family finds out about Ikura’s motivation, she simply shifts in line 4 to “omniscient” voice and presents the explanation herself.


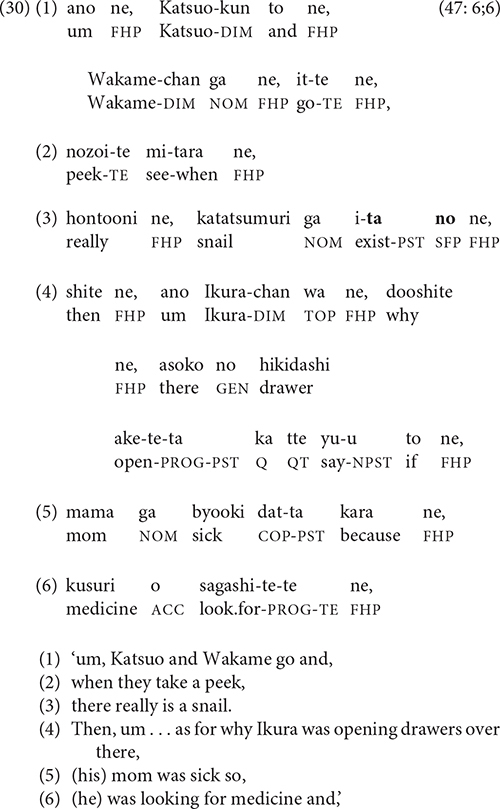


As with the referential breaks created by switch-reference, the boundaries of the narrative units in (29–30) are highlighted and strengthened by iconic breaks in the ongoing clause chain.

## To Link or Not to Link: a Statistical Analysis

The preceding qualitative analysis has identified potential motivations for linking clauses (particular semantic relations) as well as for ending clause chains (switch-reference and the boundaries of narrative units). Yet these motivations do not mandate clause linking or chain ending: narrators are free to end a chain between two clauses with a close semantic relation or to continue chaining through the end of an episode. Statistical analysis can help clarify the impact of semantic relations, switch-reference, and the boundaries of narrative units on the probability that narrators will link one clause to the next or end the clause chain in progress.

### A Mixed-Effects Model

To explore the factors that may be motivating narrators to continue or end clause chains, a mixed-effects analysis has been performed, using R (Fox and Weisberg, 2019; R Core Team, 2019), Ime4 (Bates et al., 2015) and rms (Harrell, 2019). Mixed-effect models are ideal for observational, corpus-based data, which are not well balanced and very “noisy” compared with experimental data (Gries, 2015, p. 97). For the present study, a mixed-effects model is required, since each narrator in the sample contributed more than one data point, i.e., clause. This lack of independence in the data is handled in a mixed-effects model by separating out the effects of the individual differences between narrators, i.e., “random effects,” thereby permitting a more accurate assessment of the “fixed effects,” i.e., the relationship between the predictor (independent) variables and the (dependent) variable of interest (Gries, 2015).

The dependent variable in the model is clause type: non-final or final. The fixed effects predicting clause type include: (1) Semantic Relation, (2) Switch-Reference, (3) Unit Boundary, (4) Task, (5) Age, (6) Chain Length, (7) Chain Use (percentage of chained clauses in a story), and (8) Diverse Non-final (diversity of clause-linking connectives). The random effects are varying intercepts for the speakers. The data for the analysis are the 2,428 chained clauses in the stories: 1,630 for the children and 798 for the adults.

The first three variables—Semantic Relation, Switch-Reference, and Unit Boundary—are motivated by prior research, as described in Sections “To Link: Semantic Relations in Non-final Clauses,” “Switch-Reference in Non-final Clauses,” and “Boundaries of Narrative Units,” respectively. It was anticipated that the relatively “tight” Manner and Causal semantic relations would increase the probability of non-final clauses, i.e., clause linking, while switch-reference and narrative unit boundaries would increase the probability of final clauses, i.e., ending clause chains. The effects of Semantic Relation, Switch-Reference, and Unit Boundary on clause linking vs. ending were predicted to vary by Task and Age. Since the cartoon task was simpler than the video task, the effects of Semantic Relation, Switch-Reference, and Unit Boundary were expected to be stronger in the cartoon stories. The effects of these three variables were also expected to be stronger with increasing age, as the children’s familiarity with the factors motivating clause linking and chain ending increased.

The fixed effects Chain Use, Chain Length, and Diverse Non-final were included in the model because it was anticipated that they would increase the probability of non-final clauses and interact with Age. Thus it seemed likely that with increasing age, narrators would link a higher percentage of the clauses in their stories into chains (Chain Use), include a greater number of clauses in their chains (Chain Length), and use a more diverse repertoire of clause-linking connectives in non-final clauses (Diverse Non-final). In addition to interacting with Age—and consequently with any other variable that interacted with age—these three variables were also expected to have an effect on the dependent variable of non-final vs. final clause type. That is, since Chain Use, Chain Length, and Diverse Non-final all involve linking clauses into chains, it was predicted that increases in these three variables would increase the probability of non-final clauses.

### Coding

#### Clause Type (Non-final, Final)

Clause type was coded on the basis of verb form: clauses with linking verb suffixes or finite verbs followed by connectives were coded as “non-final,” while clauses with finite verbs not followed by conjunctions were coded as “final.” Given the flexibility of word order in Japanese, non-final clauses were sometimes produced at the ends of sentences, after the “final” clause to which they were semantically linked; such clauses were coded as “non-final.” Following colloquial usage, clauses with non-final connectives but sentence-final intonation were coded as “final”; there were 20 such clauses (0.8%) in the data.

#### Semantic Relation (Causal, Comparative, Dialog, Manner, Rerun, Setting, Temporal)

Each clause was coded for its relation to the next clause, as follows:

*Causal*: physical cause, psychological motivation, or reason for next clause
*Comparative*: additive, contrastive, or concessive relation to next clause
*Dialog*: reported speech responded to in next clause
*Manner*: speech, thoughts, emotions, or actions in partial overlap or simultaneous with next clause
*Rerun*: clause is reformulated, corrected, or repeated in next clause
*Setting*: time, place, arrival of characters, or ongoing activities that set the scene for next clause(s)
*Temporal*: actions, events, states that precede those in next clause

The Temporal relation was coded only if more specific temporal relations—typically Causal, Dialog, or Setting—were not relevant. The last clause in a story as well as the last clause before the narrator left the storyline to address the interviewer were not coded for semantic relation.

#### Switch-Reference (SameSubj, DiffSubj)

Each clause was coded as having a subject referent that was the same or different from that of the following clause. Many children overused subject ellipsis but the predicate—in conjunction with the cartoon or video—usually clarified the intended subject. The last clause in a story was not coded for switch-reference.

#### Unit Boundaries (ContUnit, EndUnit)

A clause was coded “EndUnit” if it was the final clause in a setting, episode, or story; abstract or coda; or particular narrator perspective. All other clauses were coded “ContinueUnit” (ContUnit) since they belong to the same narrative unit as the following clause.

Episode boundaries were identified on the basis of shifts in time, place, characters, and ongoing activity in the input story. The Sazaesan video was analyzed as having 11 potential episode boundaries—of which most narrators recounted a subset—and the cartoon stories as having three: frames 1–2 (setting), frames 3–5 (episode 1: play on swings and slide, loss of balloon), and frames 6-9 (episode 2: exchange of balloon for hat, departure).

Following Labov (1972, pp. 363–365), abstracts were defined as introductory summaries of story characters and/or plot, while codas were defined as the narrator’s concluding commentary, minimally *sorede owari* ‘and then (it was) the end.’ In stories with codas, both the last clause of the final episode and the last clause of the coda were coded “EndUnit.”

#### Speaker

Each clause was assigned a number from 1 to 70 representing narrator; 68 narrators who produced at least one story with at least one clause chain were included in the analysis.

#### Task (Cartoon, Video)

Each clause was coded for narrative task.

#### Age

Each clause was coded for the narrator’s age group (see Table 1).

#### Chain Length

Each chain in a narrative was assigned a number corresponding to its order within a story. To measure Chain Length, each clause was given two numerical codings: its chain number and its sequential position within the chain. For example, one 5-year-old started his narrative with a two-clause chain: *Buranko to suberidai ga atte ne, Yukichan to Satchan ga asobi ni kita no.* ‘There were swings and a slide and, Yukiko and Sachiko came to play.’ The clause chain number was coded as “1” for both clauses, since this was the first clause chain in the story. In a separate coding, the first clause was coded “1” and the second “2” as the first and second clauses, respectively, within that first chain. Using these two codings, the number of clauses within each chain in the sample was calculated.

#### Chain Use (Percentage of Chained Clauses)

Chain Use was calculated as the number of clauses in a story that were linked into chains (including the final clauses of chains) divided by the total number of clauses in the story.

#### Diverse Non-final

This variable represents the diversity of clause-linking devices in the non-final clauses of a story. In the cartoon and video stories, a total of 43 clause-linking devices were used, including particular clause-linking verb suffixes, conjunctions following inflected verbs, and a few instances of gapped or ellipted verbs and of the stem (*renyookei*) form of the verb. Using the equation for Shannon entropy, H was calculated for every story and the value assigned to each clause in the story. The cartoon and/or video stories of 26 children have a Diverse Non-final value of 0, since every non-final clause in the story has the same clause-linking form, usually -*te*. The value of H increases as the number of different clause-linking devices in the story increases and as they become more equiprobable. Thus a story with 23 connectives, 22 of which are -*te*, has a score of 0.258, while a story with nine connectives—seven -*te*, one -*tara*, and one *tte*—has a score of 0.986. The values of H range from 0 to 3.09.

### Results

A multifactorial, mixed-effects model was fit, following the stepwise model selection procedure in Zuur et al. (2009) and Gries (2015): variables that are not significant or do not participate in any significant higher-order interactions are eliminated. The final model, summarized in Table 4, is significant: LR = 262.33, df = 12, *p* < 0.0000001. Each of the three main fixed effects—Semantic Relation, Switch-Reference, and Unit Boundary—has a significant effect on Clause Type in the predicted direction. The semantic relation Manner strongly increases the probability of non-final clause type, i.e., clause linking, but counter to expectation, the Causal relation does not have a similarly powerful impact. Unit Boundary and, much less strongly, Switch-Reference increase the probability of final clause type, i.e., chain ending. Task interacts significantly with Unit Boundary: there is a higher probability of ending clause chains at the boundaries of narrative units in the cartoon stories. As Chain Use (the percent of linked clauses in a story) increases, so does the probability of non-final clauses (necessarily), but the expected increase with Age was not found. Contrary to prediction, as the diversity of clause-linking devices (Diverse Non-final) increases, the probability of non-final clauses decreases. Chain Length and—most surprisingly—Age did not have significant effects on Clause Type or participate in any higher-order interactions in the best-fitting model.

**TABLE 4:** Results of model (predicted level of Clause Type = ‘Non-final’).

**Random effects:**				

**Groups Name**	**Variance**	**Standard**		
		**Deviation**		
Speaker (Intercept)	0.068	0.261		
Number of obs: 2235	groups:	Speaker, 68		

**Fixed effects:**	**Estimate**	**Std. Error**	**z value**	**Pr(>|z|)**

(Intercept)				
UnitBoundEndUnit	−0.600	0.348	−1.727	0.084.
SwitchRefSameSubj	−1.544	0.243	−6.343	2.263–10 ***
SemRelComparatv	0.306	0.133	2.304	0.021 *
SemRelDialog	0.581	0.257	2.262	0.023 *
SemRelManner	0.336	0.257	1.307	0.191
SemRelRerun	2.098	0.368	5.674	1.39e−08 ***
SemRelSetting	−0.126	0.165	−0.371	0.711
SemRelTemporal	−0.164	0.166	−0.988	0.323
TaskVideo	0.459	0.182	2.522	0.012 *
DvrsNfmal	−0.477	0.088	−5.424	5.83e−08 ***
ChainUse	2.820	0.383	7.370	1.70e-13 ***
UnitBoundEndUnit: TaskVideo	0.629	0.302	2.082	0.037 *

Signif. Codes: 0 ‘***’ 0.001, ‘**’ 0.01, ‘*’ 0.05, ‘.’ 0.1, ‘ ’ 1.

Although significant, the model has a relatively low degree of discriminatory power: C = 0.76. The fixed effects account for only 26% of the variance in the data (*R*^2^marginal = 0.26), to which the random effect, Speaker, does not add much (*R*^2^conditional = 0.27).

**FIGURE 1:**
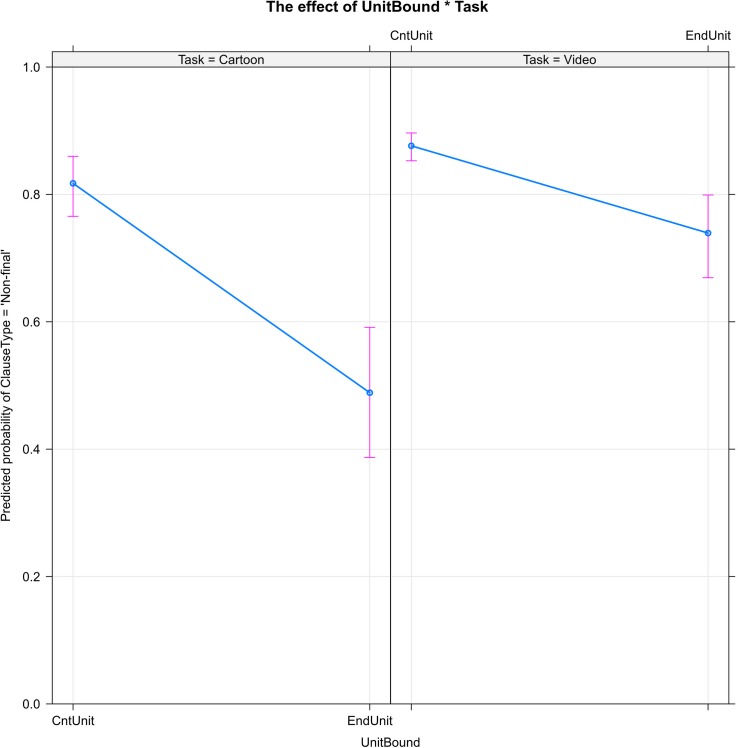
The boundaries of narrative units (UnitBound) decrease the probability of clause linking (ClauseType = ‘Non-final’): the percentage of non-final clauses is higher within narrative units (CntUnit) than at the ends of units (EndUnit). The effect of narrative unit boundaries is stronger in the cartoon task than in the video task.

Figure 1 shows the significant effect of narrative unit boundaries (UnitBound) on the use of non-final vs. final clause type. In both cartoon and video stories, the probability that narrators will use a non-final clause decreases—i.e., their probability of using a final clause increases—when they reach the end of a narrative unit such as a setting, episode, or shift in narrator perspective.

Of the predictor variables, UnitBound is the only one to interact significantly with Task. The effect of narrative unit boundaries is stronger in the cartoon task: the end of a narrative unit reduces the probability of continuing a clause chain by more than 30% in the cartoon stories, but only by about 15% in the video stories.

**FIGURE 2:**
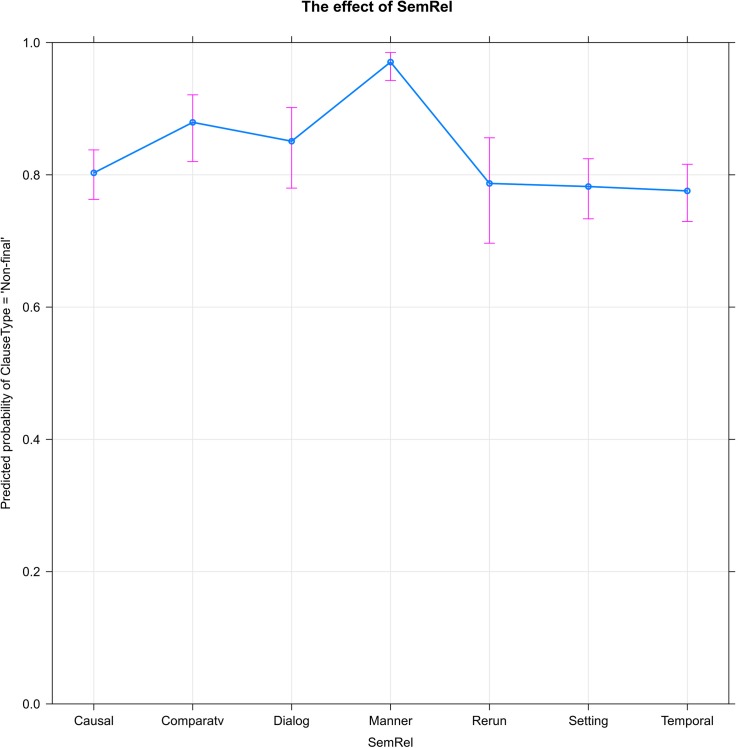
Of the seven semantic relations coded, Manner strongly increases the probability of clause linking (ClauseType = ‘Non-final’).

Figure 2 shows the significant effect of semantic relations (SemRel) on the probability of clause linking (ClauseType = ‘Non-final’). The Comparative relation, which encompasses additive, contrastive, and concessive relations between clauses, is associated with a slight increase in clause linking, as (to a lesser extent) is Dialog, the relation between successive turns in reported dialogue. But it is the Manner relation that stands out as strikingly different from the others, strongly increasing the probability that the narrator will link the current clause to the following.

**FIGURE 3:**
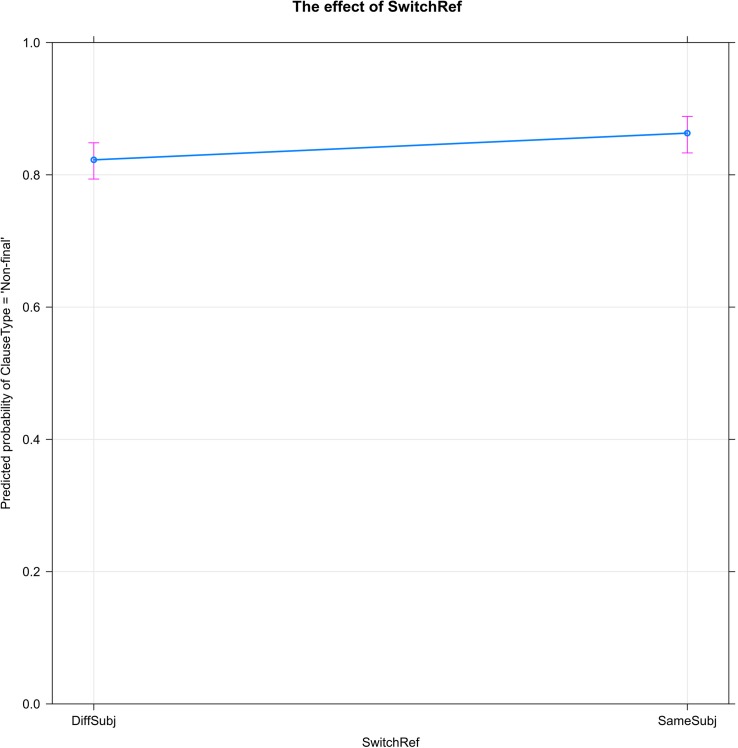
Switch-Reference (SwitchRef) decreases the probability of clause linking (ClauseType = ‘Non-final’): the percentage of clause linking is lower when the subject referent is changed (DiffSubj) than when the same referent is maintained (SameSubj).

Figure 3 shows the small, though significant, effect of switch-reference (SwitchRef) on clause type (ClauseType = ‘Non-final’). As predicted, switching to a different subject lowers the probability that the narrator will link the current clause to the following, while maintaining the same subject increases the probability of clause linking.

**FIGURE 4:**
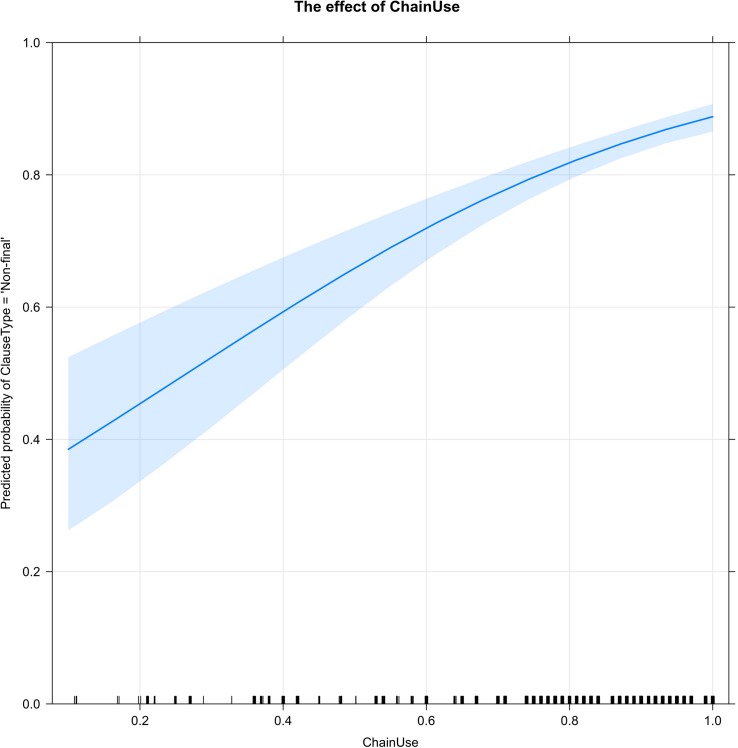
An increase in the percentage of chained clauses (ChainUse) in stories necessarily increases the probability of clause linking (ClauseType = ‘Non-final’). The ticks along the *x*-axis show that the densest concentration of stories is at the high end of the scale: 74–100% chained clauses.

Figure 4 shows the effect of Chain Use (the percentage of clauses linked into chains) on clause type (ClauseType = ‘Non-final’). As the percentage of chained clauses in a story increases, the probability of non-final clauses (necessarily) increases. The densest concentration of stories is found at the high end of the scale, where about 74% or more of the clauses in a story are chained and non-final clauses are highly predictable.

**FIGURE 5:**
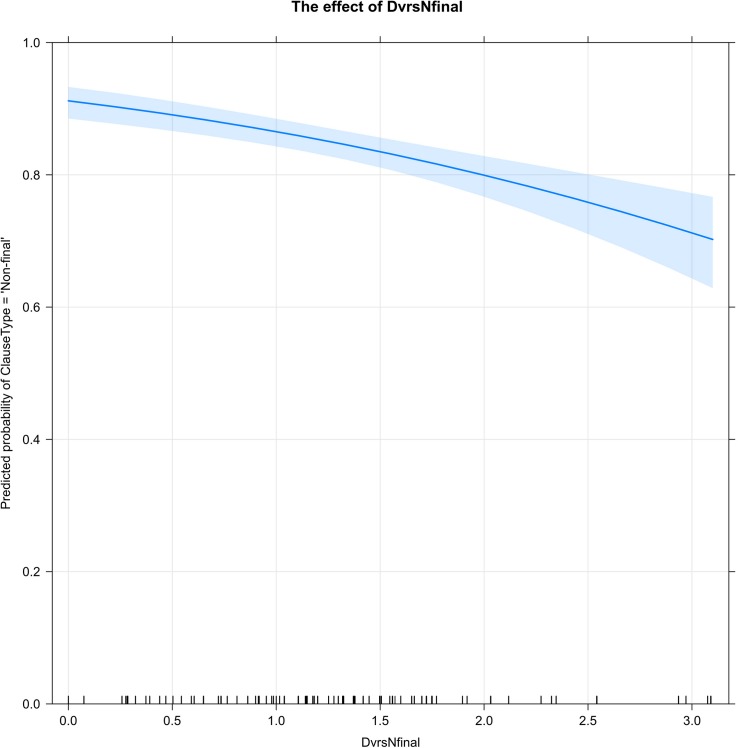
The diversity of connectives in non-final clauses (DvrsNfinal) is represented by values of *H* along the *x*-axis. As the diversity of connectives increases, the probability of clause linking (ClauseType = ‘Non-final’) decreases. The ticks along the *x*-axis show that most stories have a high (80–90%) percentage of linked clauses and a moderate (0.25 – 1.75) diversity of connectives in non-final clauses.

As with Chain Use, it was anticipated that a greater diversity of linking forms (DvrsNfinal) would increase the probability of non-final forms. However, as Figure 5 shows, very high levels of diversity in clause-linking forms are actually associated with a decreased probability of clause linking.

The highest percentages of non-final clauses are found in stories with little or no diversity of non-final forms; this typically occurs when the narrator relies very heavily or exclusively on the default connective -*te*. As the diversity of clause-linking forms increases, the percentage of clause-linking decreases; the lowest rates of clause-linking are found in stories with the highest levels of non-final diversity. As the ticks along the *x*-axis indicate, this pattern is comparatively rare: most stories have a high percentage (80–90%) of linked clauses and a moderate diversity (0.25 – 1.75) of non-final forms.

### Discussion

The statistical analysis in this study is, to my knowledge, the first attempt to model the functional motivations underlying clause chaining in narrative discourse. As such, it has a dual goal: to discover which potential semantic and discourse predictors play a significant role in clause linking/chain ending and to demonstrate that a statistical approach—in particular, mixed-effect modeling of corpus data—can shed light on the semantic and discourse functions of clause chaining.

The significant fixed effects in the model—Semantic Relation, Switch-Reference, Unit Boundary, Chain Use, and Diverse Non-final—give insight into the forces that motivate speakers to link clauses into chains and to end chains in progress. The semantic relation of Manner, which typically links characters’ thoughts, feelings, and speech to their actions, is a strong motivator of clause chaining, but the Causal relation lacks comparable impact. When narrators switch the subject referent or reach the end of a major narrative unit, the probability increases that they will end their current clause chain. The probability of non-final clauses necessarily increases as narrators’ percentage of linked clauses (Chain Use) increases, but decreases with the diversity of their connectives. The relatively weak predictive power of the model implies, not surprisingly, that these are not the only factors responsible for continuing and ending clause chains during narration.

For this analysis, only the boundaries of the highest-level narrative units were coded: between setting and episode, between episodes, and between different narrator perspectives. Including episode-internal shifts might have strengthened the effect of Unit Boundary on chain ending. In these stories there is no one hierarchical level at which even a single narrator will consistently end clause chains: narrators often ignore an episode boundary only to end the clause chain at a minor, episode-internal shift. This kind of inconsistency, both across and within narrators, means that the ends of clause chains will never correspond exclusively to the highest-level narrative units.

The interaction of Task with Unit Boundary probably reflects the different cognitive demands posed by narrating from the cartoon vs. the video. Telling the video story was much more challenging, requiring narrators to constantly retrieve material from memory, whereas the cartoon stories were told while viewing the pictures. Boundary cues such as changes in location and characters are depicted simply and clearly in the cartoon but presented dynamically in the video, where they must be processed quickly to keep up with the storyline. The relative simplicity of the cartoon task may have allowed narrators to devote more attention to marking the ends of narrative units by ending clause chains.

Although temporal sequence is generally regarded as the hallmark of clause chains (Iwasaki, 2002, p. 261), the semantic relation with the strongest impact on clause linking, Manner, involves simultaneity or temporal overlap rather than strict sequentiality. Against the backdrop of primarily temporal/causal sequential relations in their stories, the narrators apparently felt the closest connection between simultaneous interclausal relations.

Unexpectedly, causal relations did not elicit a higher probability of clause linking than temporal ones, perhaps because the internal composition of both relations, as coded for this analysis, is rather heterogeneous. Causal relations included very tight bonds of physical causality between events, more loosely related psychological causality (emotional responses to events that could have been different), and reasons for events that are separate in time and place (e.g., Ikura’s mother became ill, so he was cared for at Sazaesan’s house). Similarly, the Temporal relation encompassed tightly related enabling actions that initiate—but do not cause—event sequences as well as what Chafe (1979, p. 178) calls “temporal elasticity” at episode boundaries. A more fine-grained coding of semantic relations might have yielded a stronger effect of this variable on clause type, increasing the predictive power of the model.

With respect to referential continuity, the two-protagonist cartoon and multi-character video elicited an unusually high rate of switch-reference compared to previous findings. Many stories in this sample have a different subject in almost every clause, making switch-reference a much less compelling reason for these narrators to end a clause chain. While this may have contributed to the relatively weak effect of switch-reference in the model, the Same Subject status of Manner clauses most likely played a role in the strength of that semantic relation as a predictor of clause linking.

An unexpected outcome is that the diversity of the connectives in non-final clauses (Diverse Non-final), which was predicted to increase clause linking, is instead associated with a higher probability of chain ending. The original prediction envisioned a narrator who commands a diverse repertoire of clause-linking connectives and deploys it maximally. The negative association between the diversity of non-final forms and clause linking suggests a different profile at a high rate of diverse connectives: a narrator who is sensitive not only to the semantic nuances motivating a variety of specific connectives but also to the discourse factors motivating sentence-final forms.

The most surprising result is a negative one: age is not a significant predictor of clause linking and does not interact significantly with any of the other predictors in the model. Chain length, the percentage of chained clauses (Chain Use), and the diversity of linking forms (Diverse Non-final) do not increase with age as predicted. Nor do the effects of semantic relations, switch-reference, and episode boundaries change significantly across the age range of narrators. There may well be other age-related differences; prior research on these narratives has found significant age differences in the frequency of encoding switch-reference subjects with overt noun phrases (Clancy, 1992). But whatever other developmental differences exist in the data, when it comes to continuing/ending clause chains, the narrators in this sample are responding in similar ways to the set of predictors in the model: both children and adults tend to link clauses with the Manner relation and to end clause chains when switching the subject referent or ending a narrative unit. Future research on younger children, especially longitudinal research on the clause chains produced by 2- and 3-year-olds in storytelling, is needed to shed light on the process by which they develop adult-like treatment of clause linking and chain ending.

## General Discussion and Conclusions

This study takes up the analysis of clause chaining at a stage of development when most Japanese children have probably had a year or two of experience using clause-linking and chain-ending forms. As documented in longitudinal research, children can link clauses with -*te* and inflect verbs for past tense shortly after 2 years of age (Okubo, 1967; Fujiwara, 1977; Clancy, 1985). The 3-year-olds in the present study can use -*te* to link clauses in their stories on the basis of semantic relations much like those in the clause chains of Turkish 3-year-olds: manner, causality, and temporal relations of sequence, overlap, and simultaneity (Aksu-Koç, 1994; Slobin, 1995). On the other hand, some of the youngest children in this sample struggle to produce a connected narrative rather than a series of single-clause responses to prompting.

The clause chains that the children did produce show a surprising lack of developmental change. Since age is not a reliable measure of linguistic development, the children in different age groups may well have had overlapping levels of narrative skill, arising from individual differences in the storytelling models they have experienced as well as in the cognitive capacities, such as attention and recall, potentially underlying particular patterns of clause chaining. Extensive variation across ages has been documented by Berman and Slobin (1994, p. 94), who found that certain adult “frog stories” in their corpus exhibit “a ‘chaining’ *and then, and then* type of style similar to that of school-age children.” Thus several factors may have mitigated against the discovery of significant age differences.

An intriguing possibility is that the strongest predictors of clause linking/chain ending in the model may have a relatively natural, iconic basis, making them easy for children to acquire. Haiman (1985, p. 210) and Watanabe (1994, p. 142) have suggested that manner clauses, which significantly increased the probability of clause linking in this study, may be construed as aspects of a single action performed by the same character. This interpretation fits many of the manner clauses in these stories, e.g., saying *tadaima* ‘I’m home’ while entering the house. Episode boundaries, with their multiple dimensions of change, represent such strong breaks in discourse continuity that narrators typically pause, produce fillers such as *ano ne* ‘uh you know,’ and sometimes even forget what comes next; it makes sense, then, that they also often end clause chains at these boundaries.

From a processing perspective, research on pausing and disfluencies suggests that speakers generally plan one clause at a time (Pawley and Syder, 2000). In Japanese, as in other languages, a new clause is usually preceded by pauses and fillers potentially indicative of planning. However, Japanese speakers typically produce each clause in a series of separate intonation units (Clancy, 1982; Iwasaki, 1993b; Matsumoto, 2000), a pattern that affords a number of clause-internal opportunities for planning. Since Japanese is an SOV language and non-final clauses are grammatically distinguished from final ones only by the forms suffixed to and/or immediately following the clause-final verb, speakers do not actually have to decide whether and how to link the clause in progress to the following clause until they reach the verb. This may reduce the amount of planning required before beginning a clause in Japanese, leaving the speaker free to make decisions about clause linking before or during the clause-final intonation unit.

Certain findings of this study can be interpreted as evidence that decisions about linking/not linking clauses have a measurable cognitive cost: (1) narrators’ self-correcting “reruns” of their clause-final choices (see Section “Reruns: Linking and Delinking Reformulations”), which may suggest that the ideal form was not accessed quickly enough; (2) the lower probability of ending clause chains at narrative boundaries in the more challenging video task (Figure 1); and (3) the high concentration of stories in the “comfort zone” of moderately diverse non-final forms and relatively infrequent final forms (Figure 5). The highly effective narrator can preplan upcoming interclausal connections, attending to both the semantic relations that warrant specific connectives and to the breaks in discourse continuity that warrant ending the current clause chain.

Various ways to simplify the cognitive challenges of clause chaining are available to narrators: narrating without clause chains, i.e., using only or primarily single-clause sentences (example 7), linking all or most non-final clauses with the default -*te* form (example 27), and telling all or most of the story in a single clause chain (see Section “Chain Length and Diversity of Connectives in Non-final Clauses”). Narrators who use few or no clause chains are spared allocating cognitive resources to the choice of appropriate connectives at the ends of clauses, as are narrators who rely solely or primarily on -*te*. Narrators who use extremely long clause chains can ignore discourse discontinuities and keep chaining right through changes in subject referent and the shifts in time, place, characters, and action at the ends of narrative units.

For a discourse-based approach to grammar, the findings of this study highlight the intimate connection between clause-level grammar and narrative structure. Identifying units in spoken discourse has been a long-standing focus of research (e.g., Chafe, 1979, 1994; Hinds, 1979); according to Longacre (1985, pp. 282–284), clause chains can correspond to a sentence in non-chaining languages, to a spoken paragraph, or, in the case of “endless” chains, to an entire discourse. The present study continues this line of work by demonstrating how narrative units such as episode or story are, in part, constituted by the clause chains that delimit their boundaries.

This study takes a preliminary step toward understanding the semantic and discourse functions of clause chains in Japanese, the cognitive processes underlying their production, and children’s development of clause-chaining skills. As the weak predictive power of the mixed-effects model indicates, there is still much to be learned about narrators’ motivations for creating/ending clause chains. Further developmental research is necessary, especially between about 2;6 years of age, where most longitudinal studies end, and just under 4;0, where this study begins. In addition to research on children’s production, studies of the available models for clause chains in conversational narratives and storybooks are essential. With so much to be done, it is hoped that this study will encourage further qualitative and statistical investigation of the semantic, discourse, and cognitive factors underlying the use and acquisition of clause chaining in Japanese.

## Data Availability Statement

All datasets generated for this study are included in the article/Supplementary Material.

## Ethics Statement

Ethical review and approval was not required for the study on human participants in accordance with the local legislation and institutional requirements at the time the study was carried out. Written informed consent from the participants’ legal guardian/next of kin was not required to participate in this study in accordance with the national legislation and the institutional requirements at the time the study was carried out. Verbal informed consent was obtained from the principals of the schools, in accordance with national legislation and the institutional requirements at the time the study was carried out.

## Author Contributions

The author confirms being the sole contributor of this work and has approved it for publication.

## Conflict of Interest

The author declares that the research was conducted in the absence of any commercial or financial relationships that could be construed as a potential conflict of interest.

## Supplementary Material

DATA SHEET S1 Appendix: Cartoon.

## Abbreviations

ACC: accusative
ADJ: adjective
CMPLT: completive
COP: copula
DAT: dative
DIM: diminutive
EXCL: exclamation
FHP: floor-holding particle
FS: false start
GEN: genitive
HON: honorific
IMP: imperative
INT: intentional
INSTR: instrumental
LOC: locative
NEG: negative
NOM: nominative
NPST: non-past
PASS: passive
PL: plural
POL: polite
POT: potential
PROG: progressive
PST: past
PURP: purpose
Q: question
QT: quotative
SFP: sentence-final particle
SUBJ: subject
TOP: topic.
