# Supplementary figures and images for: To Link or Not to Link: Clause Chaining in Japanese Narratives
Source: Front Psychol. 2020 Feb 7;10:3008. doi: 10.3389/fpsyg.2019.03008 (PMC7019389; doi:10.3389/fpsyg.2019.03008)

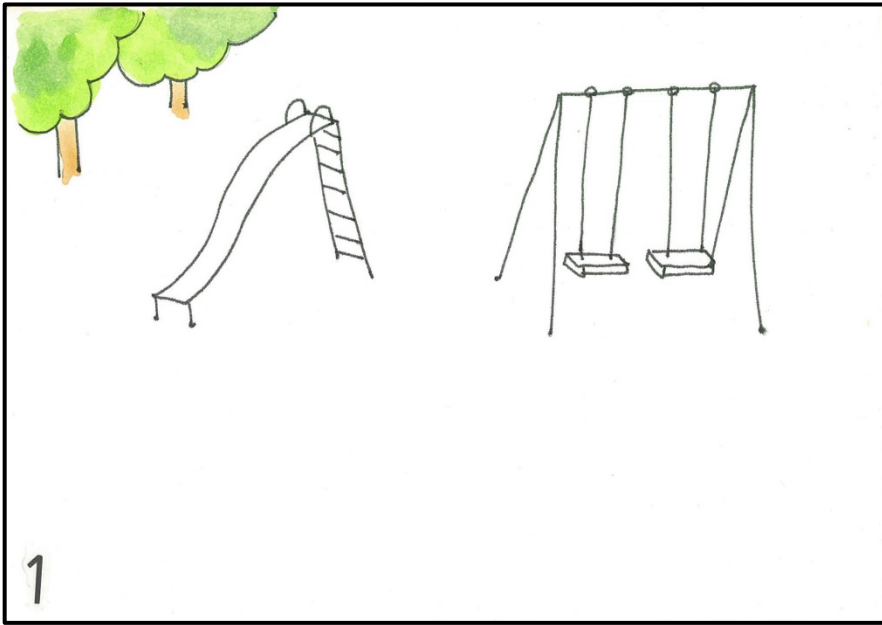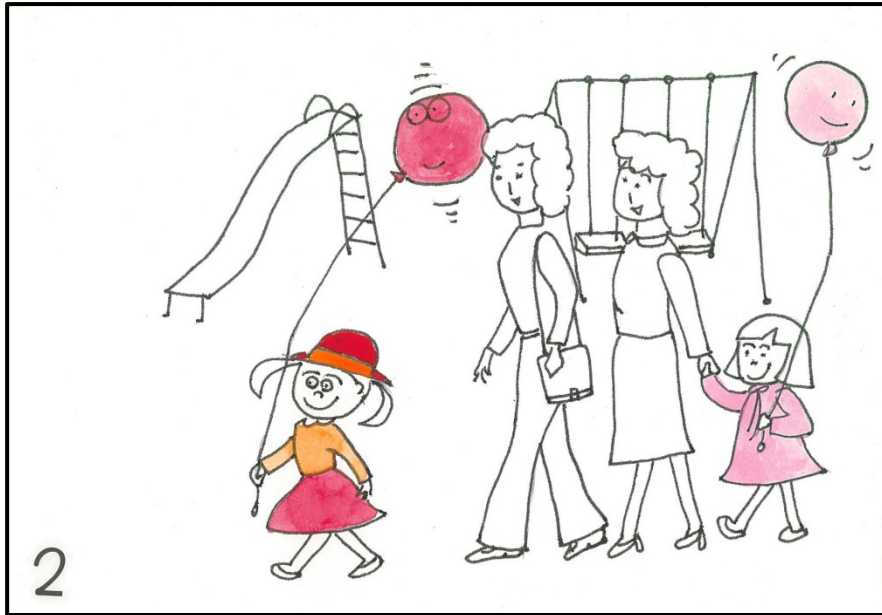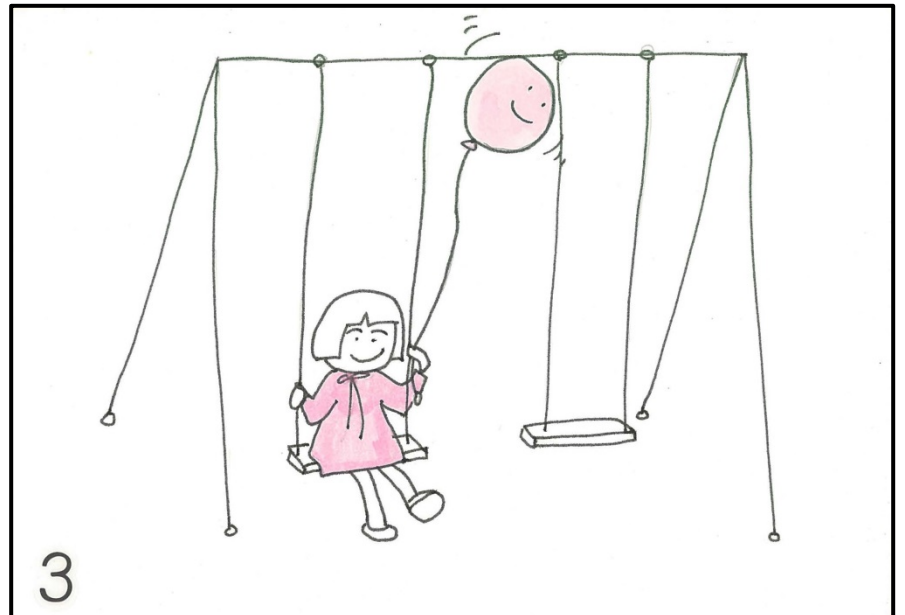

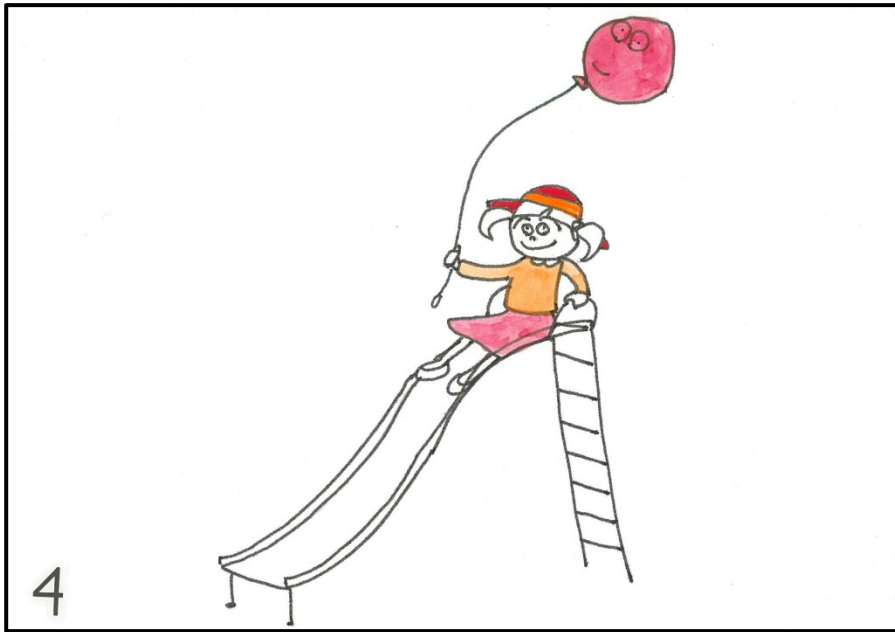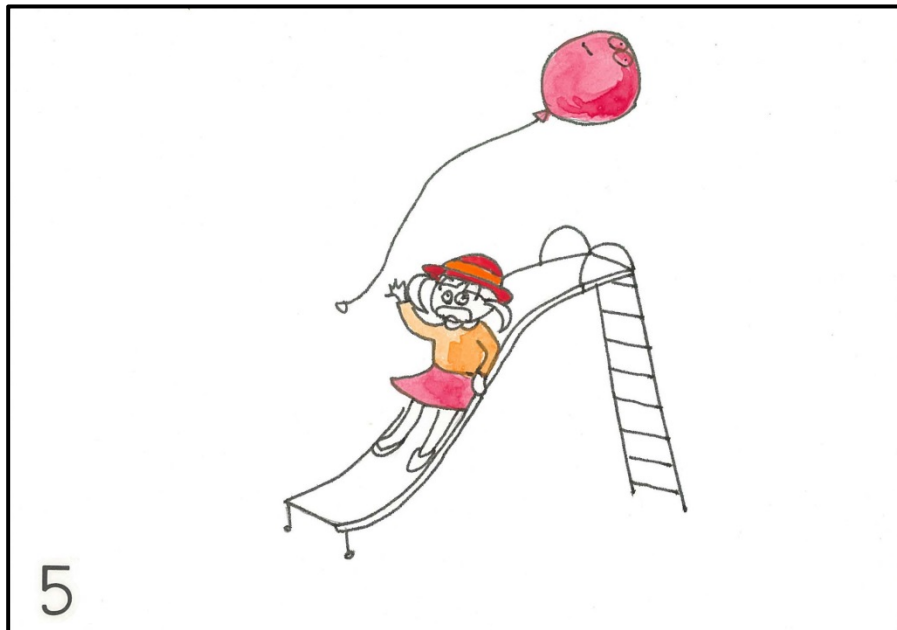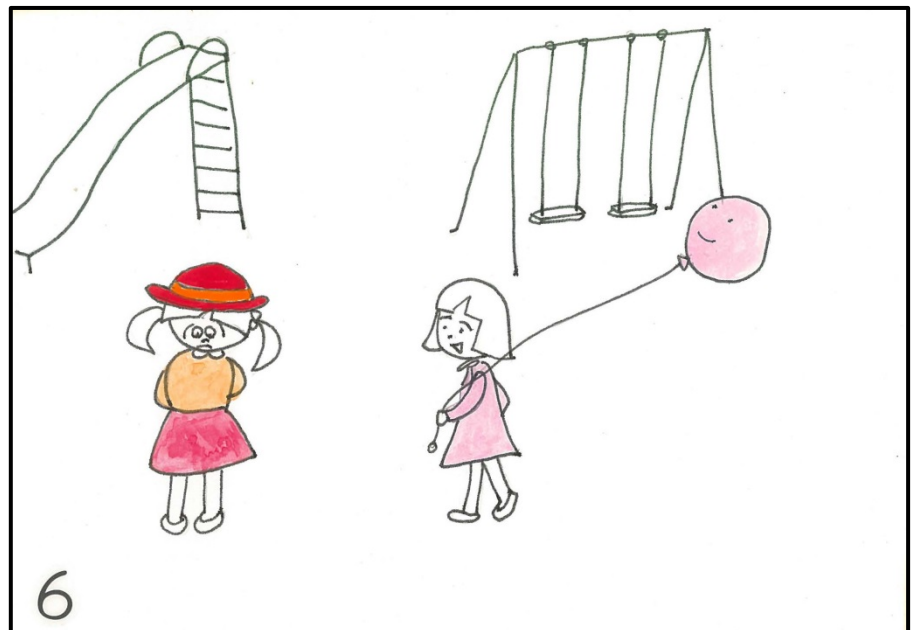

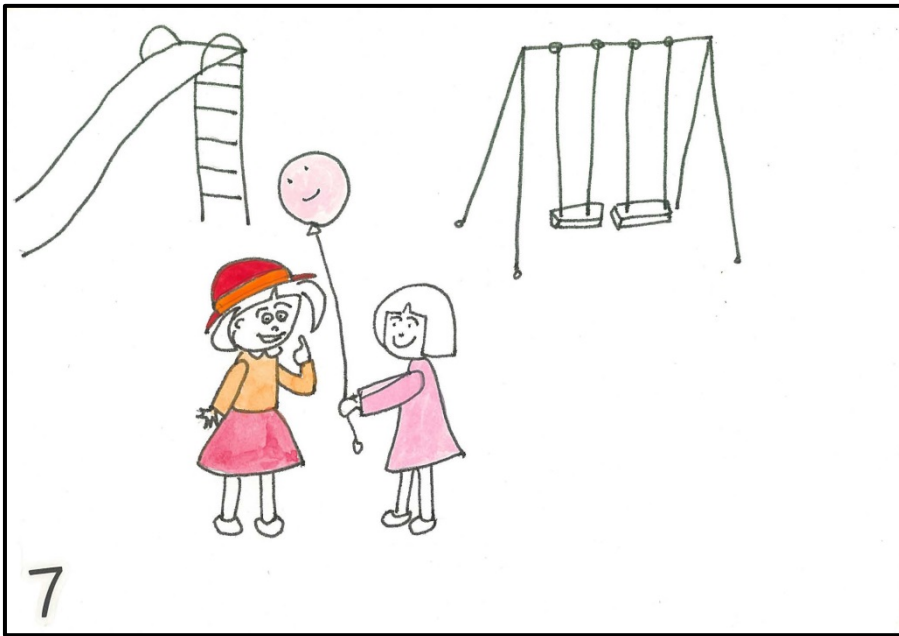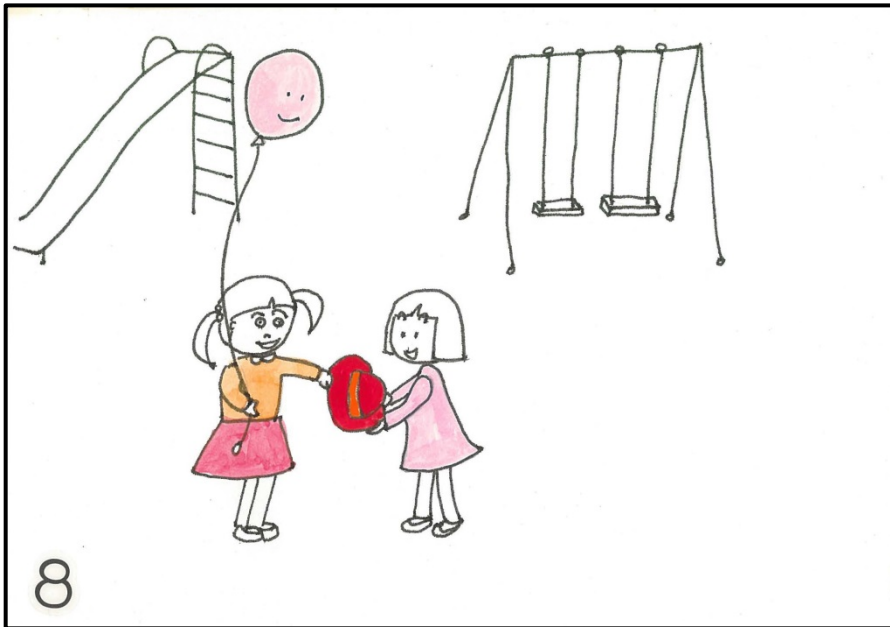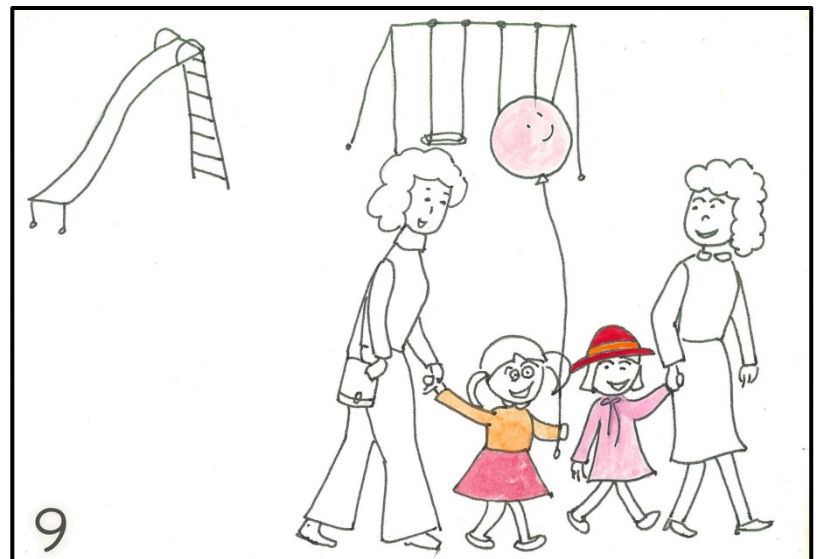

Supplement: DATA SHEET S1 — Appendix: Cartoon. [file Data_Sheet_1.PDF]
